# Smart Water Quality Monitoring with IoT Wireless Sensor Networks

**DOI:** 10.3390/s24092871

**Published:** 2024-04-30

**Authors:** Yurav Singh, Tom Walingo

**Affiliations:** Discipline of Electrical Electronic and Computer Engineering, University of KwaZulu-Natal, Durban 4000, South Africa

**Keywords:** artificial intelligence, IoT, machine learning, water quality measurement, water quality indicators, water quality sensors, wireless sensor networks

## Abstract

Traditional laboratory-based water quality monitoring and testing approaches are soon to be outdated, mainly because of the need for real-time feedback and immediate responses to emergencies. The more recent wireless sensor network (WSN)-based techniques are evolving to alleviate the problems of monitoring, coverage, and energy management, among others. The inclusion of the Internet of Things (IoT) in WSN techniques can further lead to their improvement in delivering, in real time, effective and efficient water-monitoring systems, reaping from the benefits of IoT wireless systems. However, they still suffer from the inability to deliver accurate real-time data, a lack of reconfigurability, the need to be deployed in ad hoc harsh environments, and their limited acceptability within industry. Electronic sensors are required for them to be effectively incorporated into the IoT WSN water-quality-monitoring system. Very few electronic sensors exist for parameter measurement. This necessitates the incorporation of artificial intelligence (AI) sensory techniques for smart water-quality-monitoring systems for indicators without actual electronic sensors by relating with available sensor data. This approach is in its infancy and is still not yet accepted nor standardized by the industry. This work presents a smart water-quality-monitoring framework featuring an intelligent IoT WSN monitoring system. The system uses AI sensors for indicators without electronic sensors, as the design of electronic sensors is lagging behind monitoring systems. In particular, machine learning algorithms are used to predict *E. coli* concentrations in water. Six different machine learning models (ridge regression, random forest regressor, stochastic gradient boosting, support vector machine, k-nearest neighbors, and AdaBoost regressor) are used on a sourced dataset. From the results, the best-performing model on average during testing was the AdaBoost regressor (a $\bar{MAE}$ of 14.37 counts/100 mL), and the worst-performing model was stochastic gradient boosting (a $\bar{MAE}$ of 42.27 counts/100 mL). The development and application of such a system is not trivial. The best-performing water parameter set (Set A) contained pH, conductivity, chloride, turbidity, nitrates, and chlorophyll.

## 1. Introduction

The traditional approach to water quality measurement (WQM) requires manual water sampling to determine the quality of water through analysis. This process involves the collection of water samples by humans for in situ testing by lab technicians in laboratories. While this process does not enable instantaneous WQM, it has been considered to be the most feasible solution. Research has generally been focused on improving laboratory techniques in analyzing water quality [1] and the introduction of sampling-site laboratories near water bodies to make monitoring more efficient using existing techniques [2]. It is apparent that the existing WQM techniques have shortcomings. These flaws can be broadly classified as either human error in the collection of samples, during analysis, and during the recording of data or improper lab equipment and its handling in the same processes. Furthermore, there can be cross-contamination in water samples. These methods of WQM are neither instantaneous nor timely, leading to a delayed response in the case of an emergency.

Recently, there has been an uptake on the application of wireless sensor networks (WSNs) in water quality monitoring. These methods are improving with time and keep advancing with improvements in technology and communication protocols. The WSN’s ability to capture, analyze, transmit, and display water quality data has proven to be effective and instantaneous. The WSN nodes can obtain, process, and transmit water quality parameter data instantaneously using low-power communication techniques, low-cost sensors for obtaining data, and low-power circuitry. These sensor nodes are implemented in networks; thus, a large area can be monitored with these remote sensor nodes. The deployment of Internet of Things (IoT) in WSN techniques can further improve the delivery of effective and efficient water-monitoring systems, reaping the benefits of IoT wireless systems. However, they still suffer from an inability to deliver accurate real-time data, a lack of reconfigurability, the need to be deployed in ad hoc harsh environments, and their limited acceptability within the industry.

Water quality indicators (WQIs) (namely turbidity (turb), total dissolved solids (TDSs), the potential of hydrogen (pH), total hardness (TH), chloride, fecal coliforms (FCs), electrical conductivity (EC), dissolved oxygen (DO), temperature (Temp), and oxidation-reduction potential (ORP), etc.) can be effectively determined optically, chemically, or biologically. Few of these indicators can be determined electronically, as their electronic sensors have not been developed. Electronic sensors are required for them to be effectively incorporated into the IoT WSN water-quality-monitoring system. Very few electronic sensors exist for the indicators. Table 1 indicates current water quality parameter determination techniques detailing the availability/unavailability of electronic sensors. Currently, this is the major limitation of IoT WSN systems. The unavailability of electronic sensors for some parameters has necessitated the incorporation of artificial intelligence sensory techniques for smart water-quality-monitoring systems for indicators without actual electronic sensors by relating with the available ones. These techniques are in their infancy and are still not accepted/standardized by the industry. As with any technology, it is an evolving issue, and is the subject of this work.

There have been tremendous advances in WSN water quality monitoring. Yang et al. developed and tested a wireless sensor network for monitoring an aqueous environment [37]. The system was developed due to the importance placed on developing network sensor technology in aqueous environments. Ryecroft et al. noted the major developments in the monitoring of air quality using IoT technology; however, water quality monitoring is still dependent on manual sample collection [38]. Rosero-Montalvo et al. presented an intelligent WSN system that has the ability to determine the quality of water using machine learning (ML) algorithms [39]. The aim of the research is to determine the water quality of the river through the route by creating data reports into interactive interfaces for users. Adu-Manu et al. implemented a smart river monitoring system using wireless sensor networks [40]. The focus of the system was to attain energy efficiency during the monitoring and transmission of data. Murphy et al. developed a low-cost optical sensor for water quality monitoring [41]. The development of the optical sensor was informed by the challenges that wirelessly networked sensors currently have despite advancements in water quality monitoring. O’Flynn et al. presented the “SmartCoast” multi-sensor system for water quality monitoring [42]. The SmartCoast system creates a WSN with plug and play sensors to facilitate communication with low power consumption. Seders et al. presented LakeNet [43] water quality monitoring with a network of sensors to monitor water quality in lakes and wetlands for the following parameters: temp, pH, and DO. Chen et al. developed a system with wireless transmission technology to transmit the water quality parameters of a fish farm [44]. The parameters monitored were temperature, DO, pH levels, level of the water, and the implemented sensors’ life expectancy. The system incorporates a robotic arm with a programmable logic controller, wireless transmission, and an embedded system designed to undertake automatic measurement and maintenance. Jáquez et al. developed a prototype utilizing IoT technologies in a water-quality-monitoring system (IoT-WQMS) [45]. The architecture of the system has a LoRa repeater and an anomaly detection algorithm. The results of the study indicated that the prototype improved the reliability of monitoring by promptly identifying sensor malfunctions and the increased signal range of the LoRa. Razman et al. created a water quality monitoring and filtration system controlled by Arduino [46]. The system was developed to compare the water quality of water from lake, river, and tap sources. The system monitored pH levels, turbidity, EC, ORP, and temperature through the ThingSpeak platform. None of these works present comprehensible underwater IOT WSNs that utilize ML algorithms to determine unknown parameter data using the existing data captured by sensor nodes.

The current trend in WQM introduces artificial intelligence (AI) in determining water quality. Whilst the integration of AI techniques to determine water quality is still a relatively new approach, there have been systems that incorporate them. AI techniques provide large water bodies, i.e., rivers, with greater monitoring efficiency. The substantial data collected through sensor networks can be assessed through prediction by the AI techniques. Ubah et al. implemented a system that forecasted water quality parameters using an artificial neural network [47]. The river was tested at four points for parameters that include pH, TDS, EC, and sodium. Khan and Islam presented machine-learning-based prediction and classification models to predict and classify water quality status [48]. The parameters predicted included pH, suspended solids, EC, TDS, turb, DO, alkalinity, chloride, and demand for chemical oxygen. H Aldhyani et al. implemented a water quality prediction system using AI algorithms [49]. In the system, advanced AI algorithms are developed to predict the water quality index and water quality classification (WQC). Paepae et al. reviewed the feasibility of utilizing virtual sensing for water quality assessment [50]. One of the findings of the review was that random forest, artificial neural networks, and multiple linear regression approaches dominated machine learning techniques in inferential model development. Chen et al. proposed a hybrid model of machine learning and optimization algorithms to predict water quality parameters [51]. The authors used the Adaptive Evolutionary Artificial Bee Colony–Back Propagation Neural Network (AEABC-BPNN) algorithm model, which was compared to the prediction results of support vector machine (SVM), back propagation neural network (BPNN), genetic algorithm (GA)–BPNN, particle swarm optimization (PSO)–BPNN, Artificial Bee Colony (ABC)–BPNN, and long short-term memory (LSTM) models. AEABC-BPNN was found to increase the robustness of the prediction models. The results from the testing process showed that the AEABC-BPNN approach attained convergence in 14 generations and has a quicker convergence speed. AEABC-BPNN had an optimal mean fitness of 0.0322 and it obtains prediction values that are more accurate after data anomalies are processed. None of these works provide a comprehensible solution utilizing ML algorithms to determine the *E. coli* concentrations in water using WSNs.

The work of Stoker et al. illustrated that high accuracy in predicting *E. coli* levels was possible with just five core parameters, determined through recursive feature selection: pH, DO, EC, temp, and turb [52]. The work also outlined that the inclusion of more parameters (8 or 12) only moderately increased the performance of the prediction model [52]. Stoker et al. determined that the use of a random forest (RF) model provided greater performance consistently in predicting *E. coli* levels when compared to other models [52]. Naloufi et al. found that the prediction of microbial quality in surface waters still proves to be difficult; thus, the concentrations could not be predicted in all contexts. Developing models to adapt to environmental changes was determined to be necessary [53]. Whilst the works of Stocker et al. and Naloufi et al. utilize ML algorithms to predict *E. coli* concentrations in water, none of these works utilize WSNs in the study.

There is tremendous research and advances in WQM, wireless water technologies, hardware equipment development, and data analytics. The communication technologies have acquired more rapid transmission rates, increased power efficiency, greater network support in remote areas, and have become more cost efficient in implementation in smart water quality monitoring (SWQM). These advances bring impressive gains within SWQM. Though they have their shortcomings, like power consumption, adapting to the technology outweighs their shortcomings and increases system performance in other areas. Advances in the reliability of communication technology make remote monitoring more efficient and achievable. The main areas of advances in hardware can be observed in sensor technology and energy-harvesting technology. Utilizing and developing these new sensor technologies creates larger sensor networks and enables the remote monitoring of more WQIs. The greatest data analytical advance has been the introduction of AI in determining water quality and WQI. AI techniques have provided a method of analyzing data collected from SWQM systems and identifying the status of water quality. The inclusion of AI techniques makes it possible to monitor different WQIs that do not have sensors available to remotely monitor them based on parameters that were remotely monitored through sensors. Algorithms and mathematical models have also been utilized to predict trends in water quality; thus, this allows for the prediction of future water quality changes. Whilst all of the advancements listed have been implemented in systems, there has been a lack of SWQM systems that can collectively utilize these advancements.

This work presents a smart water-quality-monitoring framework featuring an intelligent IoT WSN monitoring system. The system uses AI sensors to account for indicators without electronic sensors, as the design of electronic sensors is lagging behind monitoring systems. Whilst the use of AI sensory techniques can be applied to different water quality parameters, this study focuses on using AI sensory techniques to predict *E. coli* concentrations. The work explores WSN-based WQM systems, WSNs, and wireless sensors, as well as the constraints and challenges associated with these WSN-based systems, and the deployment of ML algorithms in the prediction of *E. coli* concentrations using parameter data from WSNs. The dataset for the developed AI technique in this work uses the samples collected from four water-treatment plants in South Africa. The plants used for data sampling were Vaalkop, Klipdrift, Wallmansthal, and Cullinan. The data were sampled over a period of 7 years (July 2011–June 2018) [54]. The aim of this study is to determine the effectiveness of using machine learning algorithms to determine *E. coli* concentrations in water and the effectiveness of using different parameter sets in machine learning models to predict *E. coli* concentrations. The chosen parameter sets were based on the cost of wireless sensor procurement and local availability of wireless sensors.

## 2. Smart Water Quality Monitoring (SWQM)

### 2.1. Smart Water Quality Monitoring (SWQM) Framework

**Figure 1:**
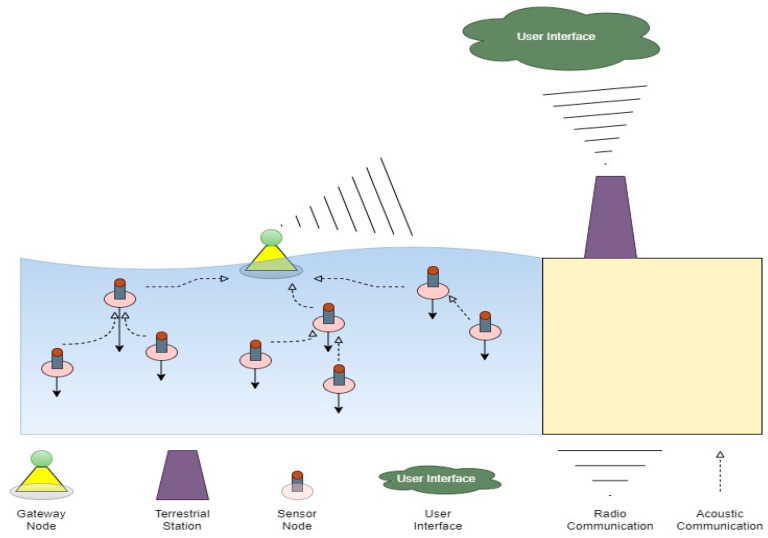
Common underwater WSN-based SWQM.

Generally, as illustrated in Figure 1, the commonly implemented SWQM models have, at a minimum, three elements that together create a basic network to monitor water quality remotely. These elements are the sensing system, the communication system, and the head end system (see [55,56,57,58,59,60,61]).

#### 2.1.1. The SWQM Sensing System

The WSN sensing system performs the collection, processing, and transfer of data. The data collection process is supported by a network of sensing devices at different locations in water bodies. This enables the sampling of water over large areas at consistent time intervals. The sensing module consists of a sensor transducer that captures the parameter and sends it to the processing unit for processing; the data are then sent through a communication unit to the intermediate nodes or gateway. All these are powered by the power supply unit. By implementing multiple sensors in various locations along the water body to acquire samples at more frequent time intervals, the accuracy in determining precise water quality levels increases. This can be attributed to more data being available for analysis when determining water quality.

When the sensing module performs the filtering and processing of data, computational devices are used to filter the data and apply algorithms to the measured parameters. Data processing can be performed using two commonly used methods [62]: in-node processing (InP) and collaborative task processing (CTP). InP involves the node using data collected from its own sensors, whilst CTP involves the nodes that are near each other sharing data with one another; thus, they use the data from different locations to perform the processing stage. The majority of WSN-based WQM systems use both InP and CTP when processing data. This allows nodes to process their own data and share their data with other nodes for enhanced or additional processing. To determine the source of contamination, InP is useful as it can provide a location based on processing at a node. To determine the general status of the water quality of a water body, CTP is more useful, as it can give an average value due to the processing of shared data.

#### 2.1.2. The SWQM Communication System

The communication system is responsible for relaying the sensed data to the head end system. This sensing node can send data directly to the gateway node in a star topology or through intermediate nodes to the gateway node, or sometimes to the cloud. The gateway node transfers data via a base station. These communication scenarios are based solely on the network topology implemented in the network, whether this is a star or a mesh topology. There are various network communication architectures available that can be implemented. The architecture can be split into short-, medium-, and long-range communication. The different remote communications implemented in various works include wired and wireless technologies: Zigbee [63], IEEE 802.15.4 [64], WiFi [65], Bluetooth [66], cellular technology, SigFox [67], LoRa [68], NB-IoT [69], and LTE M [70]. The characteristics, advantages, and disadvantages of the technologies have been extensively addressed in the literature (see [62,71]).

#### 2.1.3. The Head End System

Through the aid of remote communication, the captured sensor data are transmitted from the base station to the head end system (HES). Following the analogy of the advanced metering infrastructure (AMI) framework, The HES provides a control center with the following functionalities: data acquisition, analysis, storage, management, and the control of the whole system. It consists of the metering data management system (MDMS) that receives, stores, manages, and analyses the metering data information and events for providing better customer services. The HES further contains a user interface; additional computations are performed by the interface system: the classification and organization of the data that were obtained by the WSN. The data obtained can be stored using several methods: using offline storage solutions, online storage solutions, and/or cloud solutions. The data can be displayed to a user using tables, charts, or graphs. Additional computations can be made to illustrate water quality along water bodies by plotting maps of the water body, indicating the water quality geographically. Usually, remote monitoring stations store water quality data in databases with management systems. The databases used are mostly available online.

### 2.2. Challenges in Water Quality Monitoring

#### 2.2.1. Communication Technology

There are common challenges associated with the wired and wireless communication technologies, including coverage range, energy efficiency, bandwidth, etc. [71]. Underwater environments present communication challenges to the majority of communication technologies (such as GSM, GPRS, ZigBee, WiFi, and WiMax), as they do not propagate effectively through water. These problems include bandwidth availability, fading of signals, failure of devices, and propagation delay [72]. An effective underwater WSN-based SWQM system can incorporate underwater communication techniques with terrestrial communication techniques for the section of the system that utilizes terrestrial communication. Electromagnetic and optics communications are more constrained than acoustic communications [73]. Electromagnetic and optical transmission struggle to communicate in seawater [73] due to the conducting nature of seawater. Optic waves have difficulties with transmission distances in seawater due to their waves being absorbed by seawater. However, acoustic transmission has a stronger underwater communication ability than both electromagnetic and optic transmission due to acoustic transmission having lower attenuation in seawater [74]. Thus, the choice of communication for underwater transmission should be acoustic due to the stronger performance in seawater. Acoustic communication does have implementation challenges such as path loss, noise, multi-path, delay variance, and Doppler spread [73,75].

#### 2.2.2. Topology

Designing of underwater water quality networks provide numerous challenges. Their non-static nature adds an additional layer of difficulty when designing network topologies for underwater networks. With underwater networks relying on acoustic communication technologies, an efficient topology design would aid in negating most of the shortcomings of acoustic communication technologies. Network reliability increases with an efficiently designed network topology [73]. Energy efficiency is usually the outcome of a well-designed network topology; thus, the energy consumption issues surrounding underwater networks can be controlled [71]. Marais et al. provided an extensive review on topologies used in WSN applications [76]. The star, tree, and mesh topologies affect packet transmission and, hence, packet loss.

#### 2.2.3. Bandwidth

Efficient utilization of the accessible bandwidth in WSNs is essential for effective sharing by all the nodes in the range of the wireless network. The number of sensor nodes deployed influences the bandwidth available. The depth of node deployment affects bandwidth. The bandwidth increases with the depth of deployment of the sensor nodes [77]. The more sensor nodes there are accessing a wireless network, the lower the bandwidth available. Thus, while greater node density creates the benefit of better multi-hop routing, less bandwidth is available to the nodes as a result. Due to the utilization of energy for sensor nodes, less bandwidth is generally available to the sensor nodes for energy conservation. Bandwidth requires a balance of a suitable network topology, communication, and power consumption. An investigation into the effect of topology on network bandwidth made several findings [78]. It was found that the number of nodes in a total network affects the bandwidth of the network. Network bandwidth is affected by the number of inter-nodal links. Thus, it was recommended that, if there is a large amount of traffic in a network, only then should the number of nodes in the system be increased, and there should be fewer inter-nodal links.

#### 2.2.4. Power Consumption

Energy constraints always pose a problem in deploying WSNs. Furthermore, energy constraints exacerbate the other challenges of WQMs. The sensing system requires energy to sense, process, and transmit data. Consequently, energy consumption becomes prevalent to ensure that sufficient power is available for sensor nodes to optimally perform data capture. The total energy dissipated by the sensor node can be taken as follows: the parameter sensing undertaken by the sensor and ADC, the microcontroller and memory devices processing the data, and the communication of the data via a transceiver. The energy expended during communication of data in WSNs is much greater than the energy expended during sensing and the processing of data [79]. Generally, battery-powered sources are utilized for sensor nodes. However, in underwater sensors, battery-powered sources are rendered infeasible due to batteries being inaccessible or difficult to replace. Furthermore, charging and recharging of the systems is a constraint due to their locations. To conserve energy in a monitoring system, varying the sampling rate is a favorable method to achieve conservation. Other measures like energy harvesting have also been deployed. It is noted that WSN systems currently implemented with energy-harvesting systems mainly utilize solar panels to harvest energy from the sun. The harvested energy charges lithium-ion batteries. Solar energy harvesting for WSN networks is a popular option due to solar energy’s power density compared to other currently implemented energy-harvesting solutions [62].

#### 2.2.5. Fabrication

Underwater SWQM that use WSNs create a unique constraint during monitoring. The stress that an underwater environment exerts on sensor nodes makes them prone to water ingress and structural failure at depths. The electronic components that collectively enable the functioning of the sensor node are sensitive to liquid and will fail if they make contact with water over an extended period. Thus, the enclosure that houses these components must be waterproof and structurally resistant to the pressure exerted on them at underwater depths. Yang et al. and Ryecroft et al. fabricated enclosures to alleviate some of the challenges faced in using underwater WSNs (see [37,38]). Enclosures of sensor nodes would need to be able to withstand the underwater forces and wireless sensors would need to be waterproofed to ensure that water ingress does not damage their sensing components. Wiring would need to be protected or placed internally in the sensor node, to be protected from terrain, floating debris, and marine lifeforms. Placement of sensor nodes can create issues in communication. The closer the sensor nodes are to the bed of the water body, the greater the risk of the acoustic waves scattering or reflecting.

#### 2.2.6. Security

Security in systems that utilize wireless communication networks are a concern due to the inherent vulnerability present when there is an absence of a physical connection. In WSNs, security is compromised more significantly than other wireless communication networks due to the limited nature of the supply of energy to nodes [80]. A greater complexity in the security of a network results in greater energy usage. The data captured by sensors should determine the priority level of the security in the system. If the data captured contain sensitive information, then the compromise between energy and security would need to be addressed accordingly. If inadequate security is implemented in the system, hackers can intercept and alter data from the network. The database can also have malicious code inserted into the system. The addition of authentication and authorization protocols in the system will heighten security. Whilst current and previous works on implemented SWQM systems have very little security integration in their design, there are a few examples in simulated systems of security integration. The reason for the low number in security integration can be attributed to the increased power requirements for security protocols. In a smart solution for water quality monitoring [81], the system has integrated security protocols for data transmission. The transport layer security (TLS) was used for data encryption and JavaScript object notation web algorithms (RFC 7518) for authentication based on public/private keys. In the implemented multi-hop underwater WSN system using the bowtie antenna [38], an AES encryption module was used to ensure that all communications were encrypted.

#### 2.2.7. Theft and Damages

With the WSNs being present in bodies of water, the risk of interference caused by the nodes becomes prevalent. The disturbance caused can invite interference from human leisure (swimming, boating, etc.), wildlife activity, and natural forces (wind, etc.). It would be appropriate to ensure that the hardware utilized adheres to high quality standards and have methods available to monitor the nodes for indications of component failure or theft [80]. To lower the risk of interference, implementing sensors in a discrete manner would result in reduced attraction of wildlife. Using sensors that maintain high quality but are cost-effective would result in lower costs in replacing sensors if failures were to occur.

#### 2.2.8. The Underwater Environment

The underwater environment provides unique challenges for designers. One of these challenges is the corrosion of sensing modules, providing difficulties in the function of electronics in capturing data. When the exposed metals of sensing equipment are submerged for continuous periods, the metals react with the oxygen in the water and form metal oxides; the dissolved oxygen in the water causes corrosion. To overcome the problem of corrosion, Murphy et al. developed an optical sensor with a copper shroud. The copper shroud is corrosion-resistant; thus, it can have a longer functional lifespan underwater [41].

#### 2.2.9. Sensors

In SWQM, using WSNs, sensors are the most important component of the system as they are responsible for the acquisition of parameter data. However, these sensors have constraints that can affect their functionality, effectiveness, and their implementation. Firstly, the availability of sensors is a problem. There are wireless sensors for the measurement of water quality parameters. However, not all parameters can currently be measured by wireless sensors. This is a subject of ongoing testing, research, and development, and the commercialization of many sensors for certain parameters has not yet occurred. A solution is to integrate ML algorithms to determine the quantity of parameters. By using other known parameters that have a wireless sensor available to measure the quantity, a relationship between an unknown parameter quantity and a known parameter quantity can be established; thus, an ML algorithm can be used to predict the unknown quantity from the relationship.

Secondly, sensor calibration poses issues. Sensors can have a temporal shift in response when faced with sustained chemical and physical conditions [62]. This is known as sensor drift. Damage to the sensors caused by water or ground water fluxes can create errors in measurement by the sensors [82]. Sensor drift creates doubt in the credibility of data obtained for monitoring over a period of time; thus, developing trends or datasets from the sensor data may exhibit variation due to inconsistencies in the obtained data. Sensors must be calibrated at every specified interval to achieve accurate readings. Calibration drift occurs when there is a difference in the obtained readings from the calibrated sensor and current sensor reading in a standard solution. Calibration drift is an electronic drift and would require the sensor to be recalibrated to obtain accurate readings once again.

Thirdly, Biofouling occurs on the surface of sensors due to their immersion in water for long periods of time when capturing parameter data. Algae and bacteria cause fouling; thus, there is a high possibility of biofouling occurring in SWQM systems using WSNs. There are several factors that influence biofouling occurrence on the surface of sensors. These factors can be chemical, physical, and biological [62]. The lifespan of a sensor decreases when biofouling occurs on the surface of the sensor and there can be inconsistencies in the data obtained from the sensor, thus reducing the accuracy of the sensor. Currently, many sensor manufacturers are researching and applying new technologies to sensors, designed to reduce the effect of biofouling. However, these sensors are not currently available for commercial use.

### 2.3. Summary

Section 2 investigated the SWQM frameworks and the challenges associated with their implementation. It was found that the SWQM framework comprises a sensing system, a communication system, and an HES. The sensing system performs the collection of data, the processing of data, and the transferring of data. The communication system is responsible for relaying the sensed data to the HES. The HES provides a control center for data acquisition, analysis, storage, and management and the control of the system. The challenges associated with the implementation of SWQM were found to include communication technology, power consumption, theft and damages, the underwater environment, fabrication, security, topology, bandwidth, and sensors.

## 3. Artificial Intelligence in Water Quality Monitoring Model

In WQM, it is not possible to monitor all the water quality indicators due to the unavailability and limitations of wireless sensors. However, numerous water quality indicators are related to each; thus, using these measured parameters, unknown water quality indicators can be determined using mathematical predictive models and artificial intelligence techniques. ML algorithms create mathematical models that can predict or make decisions based on training (sample) data. They can perform these predictions and decisions, independently from human intervention. In wireless sensor networks, the use of supervised ML algorithms can greatly bolster data interpretation, classification, early warning, and water quality parameter prediction. Whilst ongoing research still faces the challenges of high power demand, the increased quality of computational resources to generate results, and the increased training data size to improve the performance of ML algorithms, the need for these techniques do outweigh the challenges. The ML techniques employed for water monitoring are presented next. This consists of the dataset and the ML models.

### 3.1. Materials and Methods

#### 3.1.1. Dataset Acquisition and Processing in the Literature

In a study by Masindi, a dataset was used in determining the status of the surface water quality for raw water in water-treatment plants [54]. Data were collected from four water-treatment plants in South Africa. The plants used for data sampling were Vaalkop, Klipdrift, Wallmansthal, and Cullinan. The data were sampled over a period of 7 years (July 2011–June 2018). The parameters sampled included pH, chloride, ammonia, sodium, turbidity, sulphate, conductivity, nitrates, color, potassium, *E. coli*, alkalinity, iron, hardness, organic carbon, precipitation potential, chlorophyll, nitrite, phosphate, and manganese. Masindi used the datasets to determine the status of the surface water quality by using the treatability index for the raw water. In this report, the sampled raw water dataset from the Vaalkop water-treatment plant was used to determine the feasibility of deploying ML algorithms to determine the *E. coli* concentrations in water.

The used dataset was the Vaalkop dataset containing 85 samples. The dataset was cleaned to exclude samples with missing data and reduced to 80 samples. The dataset was separated into two sets, namely Set A and Set B. Each set contains different parameters based on the availability or high expense of the wireless sensor required to sample the parameter. Set A contained the more commonly available and more affordable wireless sensors, whilst Set B contained the wireless sensors that are more difficult or more costly to acquire in comparison with the Set A wireless sensors. The reasoning behind the set division was to determine the effectiveness and correlation of the more commonly available or more affordable wireless sensors in predicting *E. coli* concentrations and benchmark these parameters against the Set B parameters. If the Set A parameters could provide a strong correlation to *E. coli* concentrations, then these wireless sensors could be implemented without the need for the implementation of Set B parameters in the WSN; alternatively, we might create a scenario where the Set B parameters could be implemented at a later stage in the deployment of the WSN to increase the predicting power of the system. It was also crucial to determine whether the Set A or Set B parameters were suitable for implementation in predicting *E. coli* concentrations. Parameters such as fecal coliform were excluded from testing due to the cost associated with their procurement. Locally, few WQM WSNs deploy fecal coliform wireless sensors due to procurement costs; thus, their inclusion in the study would provide little benefit, since few local systems deploy them. Various other parameters were excluded for the same reason. The parameters of Set A and Set B can be seen in Table 2. Both Set A and Set B contained pH and chloride as common parameters.

#### 3.1.2. Model Selection

Six different models were used in the evaluation: ridge regression, random forest, stochastic gradient boosting, support vector machine regression, k-nearest neighbors regression, and AdaBoost regression. Ridge regression is a regularization technique that penalizes the L2-norm of the coefficients in linear regression [83]. To improve variance and aid generalization, ridge regression aims to increase the bias. Ridge regression is usually enhanced by datasets that have a lower number of observations than the number of predictors and which have multicollinearity. The accuracy of predictions in ridge regression is improved through the lowering of the standard error. The addition of bias in predictions creates a reduction in the standard error. Thus, ridge regression will avoid overfitting during the training set to improve accuracy in the testing set predictions. A ridge regression estimator ($\hat{\beta })$ is given by Equation (1) [84].

(1) $$\hat{\beta }=\arg{min}_{\beta }{\left\|y-X\beta \right\|}_{2}^{2}+\lambda {\left\|\beta \right\|}_{2}^{2}$$

where the$\;l_{2}$—norm penalty is given by ${\left\|\beta \right\|}_{2}^{2}=$ $\sum_{j=1}^{p}\beta_{j}^{2}$. β represents the vector of regression coefficients of the markers, X is the n×p marker matrix, λ ≥0 is the tuning parameter, $y={\left(y_{1},..,\;y_{n}\right)}^{T}$ is the vector of observed phenotypes, and i, p represent markers (i = 1,2, 3,…., p) in Equation (1) [84].

The random forest algorithms use ensemble learning to provide predictions [85]. The algorithm has numerous decision trees, creating groups of random decision trees to avoid the overfitting of data. Bagging or bootstrap aggregating trains the forest. The prediction of the algorithm is the weighted average of all the decision trees. The accuracy of the prediction can be improved through the addition of more decision trees.

Stochastic gradient boosting uses an ensemble of weaker models to achieve predictions. A subsample of data from the training set is taken at random and has a weak model fitted to it [86]. This is considered to be a step approach as this process is repeated at every step to build the model. The weaker models used are often decision trees. With stochastic gradient boosting, the algorithm is quicker due to regression trees having smaller datasets fitted in each iteration in contrast to conventional gradient boosting.

Support vector machine regression (SVR) differs from linear regression. SVR searches for a hyperplane in the plane of data that will best fit the most amount of data points in a particular distance that can be achieved [87]. An advantage of this approach is the ability of SVR to contend with nonlinear correlation between the inputted variables and the prediction variable, utilizing a kernel function.

K-nearest neighbor (KNN) regression estimates the relation between the independent variables and the predicted observation through the averaging of the k-nearest observations in the neighborhood (k) [88]. The size of k is set by the user or through cross-validation. This distance between the k-nearest observations is determined using the Euclidean distance.

AdaBoost regression is an ensemble technique like stochastic gradient boosting. They both utilize and build decision trees to use as weak learners. AdaBoost uses the decision tree on the training data and will adjust the variable weights on each repeat based on prediction error, with emphasis placed on unfavorable fitting and predictions [89]. A weighted median from all the weak models will collectively provide the result of the prediction. AdaBoost reduces the loss function; thus, outliers in the data can create vulnerability in the algorithm. The weak classifiers have their performance enhanced through the introduction of reinforced training on samples that are classified as erroneous [90]. The classification error function is utilized in AdaBoost to boost weak classifier weights. The classifier can be seen in Equation (2) [90]:

(2) $$h\left(x\right)=\left\{\begin{array}{c} 1\;if\;\sum_{i=1}^{t}a_{t}h_{t}\;\geq threshold\\ 0\;\;otherwise \end{array}\right.$$

where $h_{t}$ is the weak classifier t output and $a_{t}$ is the classifier weight that is assigned in Equation (2) [90].

The decision trees of AdaBoost have one level of classification. The training dataset is weighted at each instance and the initialization of weight is given in Equation (3) [90]:

(3) weight xi=1n

where $x_{i}$ and *n* represent the training instance and the number of training instances, respectively.

Stoker et al. determined that the random forest (RF) model had superior performance in predicting *E. coli* levels in comparison to other models in the study [52]. Whilst the findings of this study indicated that the AdaBoost regressor provided better performance over the RF regressor, it still performed well enough to be considered in the implementation of the predictive model. The findings of this study indicated that the use of core parameters provided high accuracy in predicting *E. coli* concentrations.

#### 3.1.3. Evaluation Metrics

The metrics used to evaluate each of the models were root mean squared error (RMSE) and mean average error (MAE). MAE provides an insight into the accuracy of the model by ignoring the direction and measuring the average magnitude of the errors for the predictions [91]. RMSE provides insight into the standard deviation of the predicted result errors. Larger errors result in a higher value of RMSE, making it an ideal evaluation tool in models that consider larger errors unacceptable [92]. When collectively analyzing the MAE and RMSE of a model, one can identify variation in the errors of the predictions. RMSE will always be greater than or equal to the MAE measured [91]. If both MAE and RMSE are of equal value, then the errors have the same magnitude. Greater variance in the predictions will be noted when there is a larger difference between the MAE and RMSE [91]. Equations for MAE and RMSE are given by Equations (4) and (5) [93]:

(4) $$MAE=\frac{1}{n}\;\sum_{i=1}^{n}\left|x_{i}-x\right|\;$$

and

(5) $$RMSE=\sqrt{\frac{1}{n}\;\sum_{i=1}^{n}{\left(x_{i}-x\right)}^{2}}$$

where n=number of samples, $x_{i}=the\;actual\;value,$ and x=the predicted value in Equations (4) and (5) [93].

#### 3.1.4. Method

**Figure 2:**
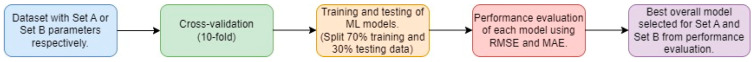
ML SWQM system model used.

All the models were implemented using Scikit-Learn and its libraries. Each model underwent a 10-fold cross-validation to achieve their best evaluation results on the dataset. K-fold validation splits the dataset into k subsets or folds. Training and evaluation of the model is completed K number of times. The validation set being a different fold each time. Figure 2 shows the system model for obtaining results for each ML model.

## 4. Results and Discussion

### 4.1. Results

The results in Table 3 represent the best evaluation results of each model. Thus, the predicted results are based on each model’s best outcome.

From Table 3, it can be observed that all the models tested provided different levels of accuracy in their prediction. On average, the random forest regressor, stochastic gradient boosting, and ridge regressor yielded the worst accuracy of the six models tested, with stochastic gradient boosting being the least accurate of the aforementioned three. The average $\bar{MAE}$ between the worst three models was 4.81 (14.50%) and the average $\bar{RMSE}$ was 10.38 (16.16%). On average, the AdaBoost regressor, support vector machine, and k-nearest neighbors showed the greatest accuracy of all six models, with the AdaBoost regressor yielding the best accuracy on average. The average $\bar{MAE}$ between the best three models was 4.19 (26.81%) and the average $\bar{RMSE}$ was 20.48 (83.24%). The large percentage of average $\bar{MAE}$ and $\bar{RMSE}$ between the best three models is due to the difference between the best two models: the AdaBoost regressor and k-nearest neighbors. The difference in $\bar{MAE}$ between the two models is 6.15 (42.80%) and 33,06 (152.07%) for $\bar{RMSE}$. The differences in $\bar{MAE}$ and $\bar{RMSE}$ between the worst-performing model on average (stochastic gradient boosting) and the best-performing model on average (AdaBoost regressor) were 27.90 (194.16%) and 59.39 (273.18%), respectively.

An observation noted was the consistency in model performance across all six models with both parameter Sets A and B. The models that performed strongly for Set A had strong performance in Set B; likewise, the weaker-performing models performed poorly across both parameter Sets A and B. This would give confidence in choosing a suitable model for implementation in WQM WSNs for *E. coli* prediction: a strong-performing model would work for both sets of wireless parameter sensors.

Table 4 contains the $\bar{MAE}$ and $\bar{RMSE}$ by adding all MAE and RMSE results, respectively, from Table 3 for parameter Sets A and B and obtaining the average for each parameter set.

From Table 4, it was determined that parameter Set A provided a higher level of performance when compared to parameter Set B. The best-performing parameter set on average was Set A. The differences in MAE and RMSE between Set A and Set B were 5.52 (21.01%) and 36.71 (89.80%), respectively. This indicated that using more commonly available wireless sensors in the initial deployment of the WQM WSN would benefit the performance of the model in predicting the *E. coli* concentrations. However, the performance difference noted was not significant; thus, the model would benefit from the addition of Set B parameter sensors at later implementations of the WQM WSN to provide more data for the model to improve its predictions.

Table 5 contains the $\bar{MAE}$ and $\bar{RMSE}$ by adding all MAE and RMSE results, respectively, from Table 3 for each of the six models used in the study and obtaining the average for each model.

From Table 5, it was determined that the best-performing model on average during testing was the AdaBoost regressor; the worst-performing model was stochastic gradient boosting. The AdaBoost regressor had an RMSE value 7.37 (51.29%) greater than the MAE value. This relatively small range in difference between the MAE and RMSE values indicates a small variance in the sample errors. The difference is relatively small when compared to the difference between the MAE and RMSE values of stochastic gradient boosting. Stochastic gradient boosting had an RMSE value that was 38.86 (91.93%) greater than the MAE value. This large range in the differences between the MAE and RMSE values indicates high variance in the sample errors. However, the largest variance can be seen in the support vector machine, with a large difference in MAE and RMSE value of 39.95 (175.68%).

There are numerous reasons that can be cited for the large variation in MAE and RMSE between the best- and worst-performing models. The main reason is the number of samples and the data quality of the dataset [94]. If more high-quality data are available, the difference between the best and worst models could be smaller. Outliers in the dataset can affect the accuracy of models, especially with linear and boosting models [95]. With boosting, outliers create issues with classifiers since they must correct previously found errors, and outliers greatly affect linear models [96].

**Figure 3:**
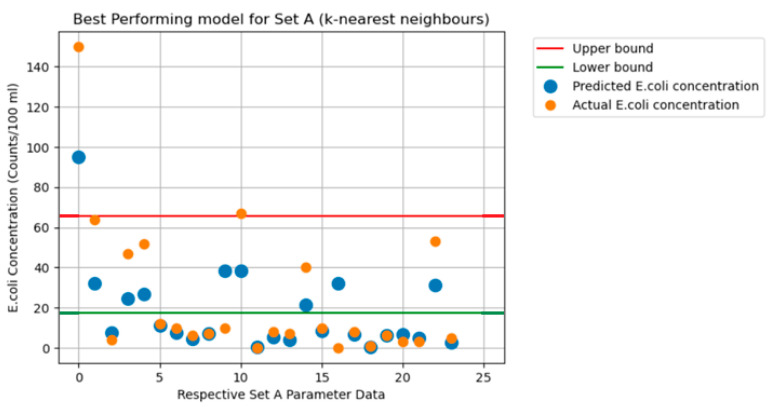
Predicted vs. actual *E. coli* concentration. (Best-performing model for Set A).

**Figure 4:**
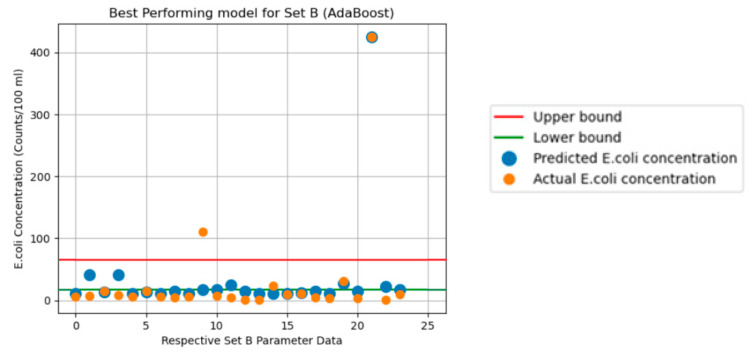
Predicted vs. actual *E. coli* concentration. (Best-performing model for Set B).

**Figure 5:**
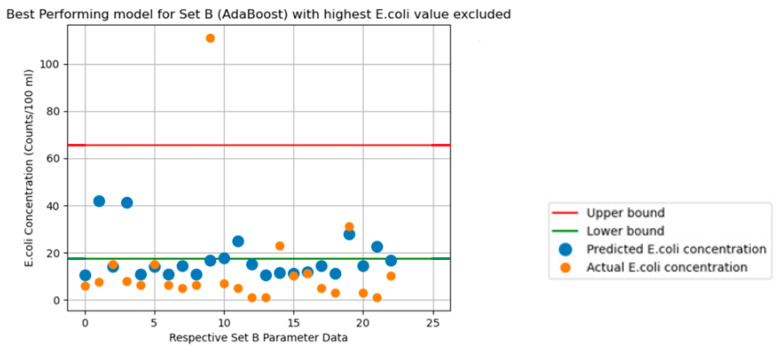
Predicted vs. actual *E. coli* concentration in Figure 4 without highest *E. coli* value.

**Figure 6:**
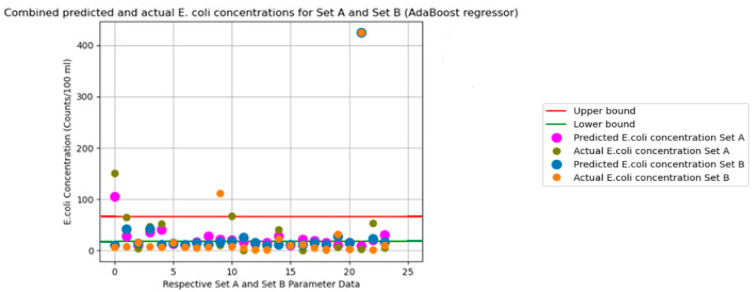
Combined Set A and Set B predicted vs. actual *E. coli* concentration. (Best-performing model overall for Sets A and B).

**Figure 7:**
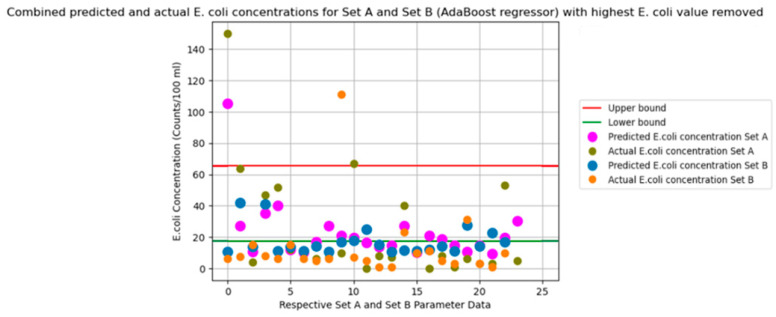
Combined Set A and Set B predicted vs. actual *E. coli* concentration in Figure 6 but without highest *E. coli* value.

The graphs in Figure 3, Figure 4, Figure 5, Figure 6 and Figure 7 illustrate the resulting data of the actual *E. coli* concentrations from the test dataset versus the predicted *E. coli* concentrations using the best-performing predictive model. Figure 3 illustrates the best-performing model for parameter Set A (k-nearest neighbors), with the red and green lines representing the 95% confidence interval upper and lower bounds for the dataset mean *E. coli* concentrations. Figure 4 illustrates the best-performing model for parameter Set B (AdaBoost regression), with the red and green lines representing the 95% confidence interval upper and lower bounds for the dataset mean *E. coli* concentrations. Figure 5 illustrates the same graph as Figure 4; however, the highest *E. coli* value (extreme outlier) from the graph in Figure 4 was excluded to improve the scale of the graph and highlight the predicting performance for *E. coli* values situated near the lower bound of the confidence interval. Figure 6 illustrates the best-performing model overall for both parameter Sets A and B (AdaBoost regression), with the red and green lines representing the 95% confidence interval upper and lower bounds for the dataset mean *E. coli* concentrations. Figure 7 illustrates the same graph as Figure 6; however, the highest *E. coli* value (extreme outlier) from the graph in Figure 6 was excluded to improve the scale of the graph and highlight the predicting performance for *E. coli* values situated near the lower bound of the confidence interval.

An encouraging observation that suits an early-warning system for *E. coli* concentration is the ability of the best-performing models to identify extreme changes in *E. coli* concentrations. From Figure 3 and Figure 4, the greatest values of actual *E. coli* concentration for each figure were noted to be 150 counts/100 mL and 425 counts/100 mL, respectively. The largest concentration of predicted *E. coli* level was also achieved on the same entry of actual *E. coli* concentration 95 counts/100 mL and 425 counts/100 mL, respectively. Whilst the best-performing model for Set A did not predict the same concentration as the actual *E. coli* level, it was still the highest predicted concentration of Set A. Thus, this would also instill confidence in currently deploying ML algorithms as an early-warning system for *E. coli* concentrations. It was observed that predicted concentrations within the 95% confidence interval dataset mean for *E. coli* concentrations had higher levels of prediction inaccuracy when compared to predictions that fell outside the bounds of the 95% confidence interval for all models.

### 4.2. Discussion

Whilst the findings of the study indicated a large difference in the accuracy of the best- and worst-performing tested models, the *E. coli* concentrations were predicted in all models with an accuracy that would suggest that the further development of models is feasible and that larger datasets of high-quality data can improve the predicting accuracy of ML models. They can become useful in early-warning detection systems for *E. coli* concentration levels. The models can be considered feasible when one considers that the stipulated acceptable range of *E. coli* concentration being 0–130 counts/100 mL (Department of Water Affairs and Forestry, 1996); the study yielded an $\bar{MAE}$ for the best-performing predictive model of 14.37 counts/100 mL and an $\bar{MAE}$ for the worst predictive model of 42.27 counts/100 mL.

As stated, the results of the study would suggest that using ML models to predict *E. coli* would be more suitable as an early-warning detection system. This was motivated by the accuracy of prediction by the best-performing ML model. The error would not instill the necessary confidence in deployers to solely rely on the prediction for their *E. coli* levels. However, if used as an early-warning detection system, relevant action can be taken, including a physical measurement to confirm and rectify the level.

The results of this study warrant further research into ML algorithms to predict *E. coli* levels; however, further work strongly depends on the availability of data to train ML models. The acquisition of reliable data from multiple sources can greatly help train models to adapt to external variations brought through ecosystems, seasonal changes, etc. It is only through continuous training of models with data that a shift to a prediction-based solution in *E. coli* detection can be the reference method of measurement. This would also apply to the prediction of other measured parameters using ML algorithms.

## 5. Conclusions

The work provides an insight into the challenges and the current and future trends of IOT WQM using WSNs. The work develops a generic IOT WQM-based framework consisting of a sensing system, a communication system, and a head end system. The work then focuses on one of the constraints of current IOT WQM systems, the unavailability of electronic sensors, and develops an AI-based framework to predict the concentrations of *E. coli* with unavailable electronic sensors. Thereby, the researchers explored the probability of predicting water quality parameters with unavailable sensors. The ridge regression, random forest, stochastic gradient boosting, support vector machine regression, k-nearest neighbors regression, and AdaBoost regression models were used in the prediction of *E. coli* concentrations. The developed results based on the MAE and RMSE performance measures indicate that *E. coli* concentrations can be predicted by AI to a fairly accurate level; however, the results indicated that the use of AI to predict *E. coli* concentrations would currently be more beneficial as an early-warning system until further research and testing can be completed, due to the level of accuracy in predictions. The results of the study indicated that the AdaBoost regressor had performed the best in predicting *E. coli* concentrations based on the performance evaluation (MAE and RMSE values). The parameters from Set A had superior performance over the parameters of Set B in *E. coli* prediction based on the performance evaluation (MAE and RMSE values). *E. coli* concentrations were able to be predicted using machine learning algorithms with reasonable accuracy. The accuracy of the predictions achieved showed that using ML algorithms would be more suitable as an early-warning detection system. *E. coli* concentrations were also predicted with parameters that can be procured with little difficultly and lower procurement costs, as seen with the prediction accuracy when using Set A parameters. Thus, the aims of the paper were achieved through the results. This work presents the possibility of developing AI techniques further to compliment parameters with non-existent sensors.

## Figures and Tables

**Table 1:** WQI measurements.

WQI	Chemical/Biological/Measurement Methods	Electronic Measurement Sensors
Turbidity	Nephelometric turbidimeter *. Secchi disk measurement *.	Analogue probes: DFROBOT SEN0189, Shanghai, China [3], YSI Xylem WQ730, OH, USA [4]. Digital probes: Aqualabo PF-CAP-C-00174 turbidity digital sensor, Champigny-sur-Marne, France [5]. Modbus industrial sensors: Daviteq MBRTU-TBD process turbidity sensor, Ho Chi Minh City, Vietnam [6].
Total dissolved solids (TDS)	Electroconductivity estimation *. Salt weighing method *. Cations and anions addition *.	Analog probes: DFROBOT SEN0244 Shanghai, China [7], Hanna instruments HI-763133, Johannesburg, South Africa [8]. Digital probes: WaterAnywhere TDS Meter TDS-3, CA, USA [9]. Modbus industrial sensors: Antratek 314990742 industrial EC and TDS sensor Modbus RTU, Nieuwerkerk aan den IJssel, The Netherlands [10].
pH	Standard Ph measurements *. Litmus papers and pH papers [11].	Analogue probes: YSI Xylem WQ201, OH, USA [12]. DFROBOT SEN0169-V2, Shanghai, China [13]. Digital probes: Tetraponics HM Digital SP-P5 pH probe, MN, USA [14]. Modbus industrial sensors: Eucatech 314990622 RS485 MODBUS-RTU industrial pH sensor, Boksburg, South Africa [15].
Chloride	Colorimetry with ferricyanide method *. Titration with silver nitrate, Mohr’s Method [16].	Analogue probes: Currently, there are no commercially available analogue chloride probes. Digital probes: YSI EXO Chloride Digital Smart Sensor (ISE), OH, USA [17]. Modbus industrial sensors: Riverplus WS102-CL, Bang Talat, Thailand [18].
Fecal coliform	Membrane filtration method *. Multiple tube fermentation techniques or pour plates *.	Currently, there are no commercially available analogue and digital sensors available. AI sensor equipment: Proteus Instruments Libelium Proteus sensor, Stoke Prior, UK [19].
*E. coli*	Membrane filtration method *. Multiple tube fermentation techniques *.	Currently, there are no commercially available analogue and digital sensors available. AI sensor equipment: Proteus Instruments Libelium Proteus sensor, Stoke Prior, UK [19].
Conductivity (EC)	Conductivity probes *. Relational TDS method *.	Analogue probes: DFROBOT SEN0451, Shanghai, China [20], YSI WQ-COND, Oh, USA [21]. Digital probes: Endress+Hauser Indumax CLS54D, Reinach, Switzerland [22]. Modbus industrial sensors: Antratek 314990742 industrial EC and TDS sensor Modbus RTU, Nieuwerkerk aan den IJssel, The Netherlands [10].
Algae	Chlorophyll concentrates *. Algal cell or colony counts *.	Currently, there are no commercially available analogue sensors available. Digital probes: YSI EXO Total Algae PC Smart Sensor, OH, USA [23]. Modbus industrial sensors: Apure BGA-206A, Shanghai, China [24].
Clarity	Secchi disk methods *. Turbidity measurement methods *.	Can use sensors for turbidity to determine the clarity based on the relationship between the two parameters.
Enterococci (Fecal streptococci)	Membrane filtration *. Multiple tube fermentation techniques or pour plates *.	Currently, there are no commercially available sensor probes.
Coliphages	Plaque assay methods *. Standardized methods of analysis have not been defined *.	Currently, there are no commercially available sensor probes.
Temperature	Devices such as thermistors, thermocouples, and thermometers *. Conductivity–Temperature–Depth (CTD) meter *.	Analogue probes: YSI Xylem WQ101, OH, USA [25]. Digital probes: DFROBOT SEN0511 DS18B20, Shanghai, China [26]. Modbus industrial sensors: ComWinTop CWT-T01S, Shenzhen, China [27].
Ammonia	Phenate colorimetric method *. Titration methods [28].	Currently, there are no commercially available analogue sensors available. Digital probes: Aquaread ammonia sensor, Kent, UK [29]. Modbus industrial sensors: Kacise KAN310, Xi’an, China [30].
Nitrate	Cadmium reduction and diazotization *.	Currently, there are no commercially available analogue sensors available. Digital probes: Sea-Bird SUNA V2, WA, USA [31], Aquaread nitrate sensor, Kent, Great Britain [32]. Xylem 107066 sensor for NH4, WA, USA [33].

* [34,35,36].

**Table 2:** List of set parameters.

Set	Parameters
Set A	pH, conductivity, chloride, turbidity, nitrates, and chlorophyll
Set B	pH, ammonium, chloride, nitrites, iron, manganese, phosphate, and sulphate

**Table 3:** List of best MAE and RMSE results for parameter Set A and Set B.

Evaluation Metric	Parameter Set	Evaluation Score	
		MAE	RMSE
Ridge regression	Set A	28.65	37.50
	Set B	36.67	83.26
Random forest regressor	Set A	36.63	58.14
	Set B	46.58	91.22
Stochastic gradient boosting	Set A	45.36	73.26
	Set B	39.17	88.99
Support vector machine	Set A	19.46	37.20
	Set B	26.01	88.18
k-nearest neighbors	Set A	12.08	19.04
	Set B	28.96	90.56
AdaBoost	Set A	15.42	20.14
	Set B	13.32	23.34

**Table 4:** List of $\bar{MAE}$ and $\bar{RMSE}$ for parameter Sets A and B.

	Set A	Set B
$\bar{MAE}$	26.27	31.79
$\bar{RMSE}$	40.88	77.59

**Table 5:** List of $\bar{MAE}$ and $\bar{RMSE}$ for each of the six models used in the study.

Model	$\bar{MAE}$	$\bar{RMSE}$
Ridge regression	32.66	60.38
Random forest regressor	41.61	74.68
Stochastic gradient boosting	42.27	81.13
Support vector machine	22.74	62.69
k-nearest neighbors	20.52	54.80
AdaBoost	14.37	21.74

## Data Availability

The dataset used in study is available online: [54].
